# Barriers to mental health services utilization in the Niger Delta region of Nigeria: service users’ perspectives

**DOI:** 10.11604/pamj.2013.14.159.1970

**Published:** 2013-04-24

**Authors:** Izibeloko Omi Jack-Ide, Leana Uys

**Affiliations:** 1 Faculty of Nursing, Niger Delta University, Wilberforce Island Amassoma, Bayelsa State, Nigeria; 2 School of Nursing and Public Health, University of KwaZulu-Natal, Durban, South Africa

**Keywords:** Barrier, cultural beliefs, mental health service, mental health education, Nigeria, Niger Delta, service utilization

## Abstract

**Introduction:**

There is only one neuro-psychiatric hospital for over four million people in the Niger Delta region of Nigeria. Low-income groups in urban and rural areas who access care through public mental health clinics are at greater risk of not accessing the needed mental health care. This study aimed to explored barriers that prevent people from utilizing mental health services, and to identifies key factors to increase access and improved service delivery.

**Methods:**

A qualitative study was conducted among 20 service users attending the outpatient clinic of Rumuigbo neuropsychiatric hospital. Ten participants were caregivers and 10 were clients, both having accessed services for at least one year.

**Results:**

The mean age was 37.7 years, 60% were males, 40% were unemployed and only 15% had a regular monthly income, while 65% live in rural areas. Barriers observed in mental health services use were physical, financial and cultural. These include absence of service in rural communities, poor knowledge of mental health services, stigma, transportation problems, waiting time at the facility and cost of service.

**Conclusion:**

Stigma remains a strong barrier to accessing mental health services, and extensive efforts need to be made to overcome ignorance and discrimination. Mental health services need to be provided throughout the health care system to enable people to access them locally and affordably, preventing the need to travel and promoting service uptake and treatment continuation.

## Introduction

Mental health disorders constitute the major causes of disabilities worldwide, accounting for 37% of all healthy life years lost through disease [1]. Mental illness is a disabling, chronic condition that poses numerous challenges in its management and as risk factors for other health problems [2]. It extols significant costs to the patient in terms of personal suffering, to the families as a result of the shift of burden of care and life-time lost productivity, and on the society at large^3^, [4]. In spite of the resulting burdens, less than 20% of persons with mental disorders received treatment in Nigeria, of whom only 10% utilize public mental health service and only 110% maintain follow-up treatment over a period of 12 months [1].

Knowledge of the barriers to mental health service utilization is particularly important in order to address the needs of people with mental disorders and plan appropriate service. In a review of barriers for mental health service use, Leong and Lau [5] identified four categories of barriers: cognitive, affective, value orientations, and physical. They hypothesized that the first three reflect cultural obstacles that impede an individuals’ intent to seek mental health services, and the fourth refers to practical barriers regarding the use of services. The practical barriers include a general lack of mental health awareness, cost of treatment, poor knowledge of how to access service, and waiting time [6]. Utilization of services is however affected by many interacting factors, such as individual and help-seeking preferences, access, availability and referral practices. Help seeking processes are events that occur between the point of first recognizing a problem (onset of illness), and when the patient enters a mental health service for treatment, as well as sustaining such treatment [7]. Help seeking behaviors provide critical links between the onset of mental disorders and the provision of professional support.

Nigeria has eight psychiatric hospitals to serve a population of over 150 million, eight schools of psychiatric Nursing, and twelve medical schools, with all mental health services only being provided at these institutions. Mental health services are concentrated in the southern urban areas with a few in the north and no services in rural areas. Patients required these services are referred from clinics across the country were specialists are not available. Nigeria has a ratio of mental health bed of 0.4 per 100.000 persons, 4 psychiatric nurses per 100 000 persons, 0.09 psychiatrists and 0.02 psychologists and social workers per 100 000 persons and a total public health expenditure of 5% of the country's budget [8, 9]. As the mental institutions are located in big cities, individuals may not seek mental health care due to poor knowledge of available services, accessibility, cost, and negative perceptions about health care system. The low-income groups in both urban and rural areas who access care through public mental health clinics are therefore at greater risk of not receiving the needed mental health care [10–12].

This service gap is filled by traditional, spiritual, faith healing and complementary medicine, the use of which is widespread in most communities, as lay perception of mental disorders are still rooted in super-natural belief systems, and therefore considered untreatable with western medicine [13–15]. In the mist of this competition with traditional care models, orthodox psychiatric care faces the challenge of proving its efficacy to all sectors of the society.

Recent discoveries in mental disorders etiology, as well as different treatment modalities, have led to the availability of more effective interventions for the outcome of persons with mental disorders e.g. alcoholics who seek help early from specialist treatment programs show better outcomes than those who do not [16]. Gulbinat et al [17] argues that although cost-effective treatment for more than 80% of those suffering from epilepsy has been available for several decades (i.e., Phenobarbital for less than US$5 per person per year), the large majority of patients with epilepsy remain untreated in most developing countries. However, negative views and stigma surrounding the disease, an absence of service, distance to services and poverty are some of the many barriers experienced by persons with mental disorders seeking care and sustaining treatment [18–20]. Furthermore, payment for mental health service is usually out-of-pocket, and as a result, the cost of procuring treatment contributes to the problem of under-utilization of services, even after treatment has been initiated [14, 21].

Although mental health care is important for the general population, it is particularly important for people with disorders who are poor and have to travel over long distances to access service. The Niger Delta region is home to over four million people, with mental health services only being provided at the Rumuigbo hospital in Port Harcourt, which provides in- and out- patient facilities to people with mental disorders. This study explored the barriers to utilization of mental health services by caregivers/clients accessing services at the Rumuigbo Hospital, the aim being to identify key elements in an effort to increase access and improved service utilization.

## Methods

A purposive sample of 20 service users attending the out-patient clinic of the Rumuigbo hospital was recruited with the assistant of the clinic sister by word of mouth, of whom 10 were health seeking clients and 10 were family caregivers, not related to client group, who accompanied their ill relatives to the clinic. The two groups were included to establish where they identified the same barriers to accessing care. Inclusion criteria were: both groups were 18 years and older, and who had at least a basic primary education and must have been attending the out-patient clinic for not less than a year in order to have had a thorough experience of the mental health care system. Clients were included who were not overtly psychotic as to enable them to comprehend and respond to the questions asked. Newly diagnosed patients and all those currently in the hospital were excluded from the study.

### Data collection

An informed consent to participate in the study was obtained from each participant, and all interviews were conducted by the primary author using a semi structured interview guide containing open-ended questions developed by the authors that had been refined in a pilot study with four service users. Participants were encouraged to discuss and reflect upon their experiences, and questions included issues relating to the onset of the disorder, the difficulties/challenges of accessing service and sustaining treatment, barriers to accessing care related to perceptions of illness, and their perceptions of mental health services. To limit intrusion into private households and protect confidentiality, participants were given the choice of being interviewed after their follow-up appointments at the clinic. A theoretical sampling framework was used.

### Ethics approval

Ethics approval for this study was granted by the Ministry of Health and the Ethics Committee of the Rumuigbo Neuro-psychiatric Hospital.

## Results

The study findings are presented within two categories, the socio-demographic results and the thematic analysis.

### Socio-demographic

The study samples were 10 caregivers and 10 clients who were not related to the caregivers. Of the 20 service users recruited, 60% are males (5 caregivers, 7 clients), the mean age was 37.7 years (41.6 caregivers, 33.8 clients), 40% were unemployed (3 caregivers, 5 clients), 15% had a regular monthly income (2 caregivers, 1 clients), 10% were tertiary level students (2 clients), and 65% lived in rural areas (6 caregivers, 7 clients) (Table 1).

**Table 1:** Demographic data of service users

Care givers						Clients					
No	Sex	Age	Employment Status	Education Status	Residence In/Outside City	No	Sex	Age	Employment Status	Education Status	Residence In/Outside City
1	M	52	Petty trader	Secondary	Outside	1	F	44	Public servant	Secondary	In
2	F	30	Unemployed	Tertiary	Outside	2	M	25	Student	Tertiary	Outside
3	F	42	Petty trader	Primary	Outside	3	F	30	Unemployed	Tertiary	In
4	F	56	Petty trader	Secondary	In	4	M	28	Unemployed	Secondary	Outside
5	F	62	Petty trader	Secondary	Outside	5	M	58	Petty trader	Secondary	Outside
6	F	35	Public servant	Secondary	Outside	6	M	24	Unemployed	Secondary	Outside
7	M	43	Public servant	Tertiary	In	7	F	36	Unemployed	Secondary	Outside
8	M	32	Petty trader	Secondary	Outside	8	M	48	Petty trader	Tertiary	In
9	M	40	Unemployed	Tertiary	In	9	M	21	Unemployed	Secondary	Outside
10	M	24	Unemployed	Tertiary	In	10	M	24	Student	Secondary	Outside

Residence: In/Outside City: This is used to represent the location of participants in respect to the hospital. In represent participants living in the city where hospital is located. Outside represent service users living in rural areas.

### Thematic

Many factors were found to act as barriers to mental health service utilization. The three barriers highlighted by the participants were categorized as physical, financial and cultural, with themes being identified within each as presented below in Table 2.

**Table 2:** Barriers and themes arising from interview transcripts

No.	Barriers	Themes
1	Physical	Poor knowledge of mental health service Centralised mental health service Waiting time
2	Financial	Travel distance/Transportation High cost of service Loss of productive income
3	Cultural	Stigma and discrimination Feelings of shame

**Physical barriers:** The factors relating to physical barriers to service utilization consist of the awareness of available service and the ability to access them, with three themes emerging namely; poor knowledge of mental health service, centralised mental health service and waiting time.

**Poor knowledge of mental health service:** Caregivers and clients held similar views that poor knowledge of the available mental health service, and the uncertainty about where to go for treatment being reasons for not seeking the needed care. As noted by Caregiver 1: “*We were not aware of this place and you know we felt the illness need spiritual attention like in this her case, we delayed, we delayed coming so the situation got worse and worse”*

Most clients believed that not understanding where to seek professional help interfered with obtaining appropriate treatment at the onset of their illness. Client 4 illustrates: *“When my illness started my father was not aware of this place, I was taken to a spiritualist. They didn't bring me here they took me to some churches and other places before we were directed to come here.”*

**Centralised mental health service:** Caregivers observed that during psychiatric emergencies i.e. relapse or adverse drug reactions where prompt attention was required, they relied on locally available sources for support (spiritual healers) due to the absence of mental health service outside the Rumuigbo hospital, this being particularly problematic for those living in rural communities as indicated by Caregiver 4. *“Please provide this type of hospital in other places to help us get the treatment. It's hard to come here from that is why we are unable to come as expected and it's affecting him, today he is fine, tomorrow is something else.”*

Clients were of the view that due to their helpless states during times of relapse, they are at the mercy of where ever their family members decide to take them for treatment. However, Client 9 believed that the proximity of service will enhance treatment outcomes. *“The government should provide this type of hospital around us at least in every local government area’ like in my place there is no hospital like this and that is why it's very difficult to keep the appointments - let the government come to our aid.”*

**Waiting time:** Long waiting time for mental health service at the clinic were raised as a significant issue by both clients and caregivers, and concerns were expressed regarding the lack of staff. Caregiver 8 complained that *“Whenever you come for clinic appointment you can't do any other thing, in fact the whole day is wasted. By the time you finish with the doctor your ordeal just begins, to get the prescriptions from that place is the greatest headache. Some of us came from very far places, we want to get back on time.”*

Clients 1 observed that the delay had a negative effect on her work as well as her mental health well-being. “The delay makes me anxious because I may not be able to go back to work today. Those of us working should be treated differently I wish we are given urgent attention to enable us go back to work”

### Financial Barriers

This consisted of the economic implications of obtaining mental health service, three themes were observed, travel distance/transportation, high cost of service and loss of productive income.

**Travel distance/transportation:** Clients observed that the long journey to access services was cumbersome and costly, and that the lack of money for transport to the hospital meant that they cannot always access the care needed. One of the caregivers (6) reported she could not afford the cost of transporting herself and ill relative to the hospital for follow-up appointments as required, and that led to a relapse in the relatives’ mental state. *“It is hard to come as the doctor said because of transport sometime we are unable to come for two, three months, sometimes even more’ at times when I don't have money to come the illness will start’ without the drugs he misbehaves, you see him on the street, destroying peoples properties which I don't want.”* Client 7 reported: *“I came by boat from yesterday and spent the night to enable me come early today. It is very hard to come most of the time due to the distance.”*

**High cost of service:** Both groups felt that their poor financial circumstances result in their inability to utilize mental health service. Client 2 illustrated: *“It has been tough for my dad and he has been taking care of all the expenses, my drugs and other things that I may need and this illness has added to his financial burden I may say.”*

Caregivers suggested that services should be made more affordable or totally free, particularly for the low-income group, to reduce the burden of the illness on their families. Caregiver 3 reported that: *“I think to meet up with these expenses is not easy- the problem here is that there is no specific amount, whatever you are asked to pay that's it, like today I was not expecting this N8,000 (US $55) for his drugs for a month supply. You will spend what you don't expect - I was not expecting that kind of amount but what can I do.”*

**Loss of productive income:** Most of the caregivers indicated that accompanying ill relatives for clinic appointments meant that they had to leave their work, trade and vocation, which resulted in loss of income due to their absence from work. Caregiver 8 indicated that: *“The impact of his illness is so much, this illness inconvenience my work and business, right now as I'm here people are calling me on phone to come over for some business issues but right now I cannot go because of him.”*

Most of the clients are unemployed and depended on family members’ support with care, and Client 3 indicated that he lost his job due to frequent applications for times off duty to attend clinic appointments. *“It's my family that is doing everything for me it's not easy with them, to buy the drugs, feed and also come for appointments. I was working but lost the job due to my illness, my boss said every time I'm going to hospital.”*

### Cultural Barriers

Community members held negative perceptions about mental disorders that resulted in families and affect persons experiencing stigma and feelings of shame.

**Stigma/discrimination:** Participants observed that due to the public's negative perception, information about mental disorders was considered too intimate to share with people outside of the nuclear family without attracting stigma. As illustrated by Client 1: *“When I was hospitalized here, I got a sick letter from the teaching hospital to my office rather than getting one here because of what people will say, the stigma so because of that you don't want to tell people, you don't, I don't tell.”*

Most caregivers’ believe that the belief associated with mental disorders as being due to wicked acts makes many families conceal the illness to avoid community gossip and rejection. This hinders appropriate help-seeking behavior of many families. As one of the caregivers (9) observed: *“We took him away from home since this illness started and his community people are not aware of his illness, it's better to keep him away from them.”*

**Feelings of shame:** Caregivers and clients held similar views regarding, with feelings of shame of being seen in the Rumuigbo hospital as being one of the main barriers to accessing services. Psychiatric hospitals were considered to be places for who are crazy, mad, or lunatics, and therefore an abominable place to be seen. An expression from a client (3): *“The outpatient clinic I would have love them to have some little privacy, not to keep everybody in that open hall like one boy that just saw me. I feel very shameful when people say all sorts and call you names.”*

Caregiver 4 also observed that the fear of being seen by friends and neighbours makes many service users opt out of treatment. “I feel shameful about my son's illness, especially if we are coming here, I don't want anybody I know to see us'That is why most times it's hard for me to bring him for treatment”

## Discussion

The fact that so many were from rural areas, two were students, more unemployed from the client group, people should be given drugs for six months to tide them over once patient is stabilized or referred to local facility for repeat prescriptions. Access to early mental health care and sustaining the treatment is critical to promote mental health well-being, as well as to identify mental health issues and prevent the disease progression. Good mental health care is important for the general population, but particularly important for individuals and families living with mental disorders.

### Socio-demographic

While there is a perception that access is a problem for those from outside the city, 65% were not city residents, indicating that despite the difficulties, people were accessing the services. Two clients were undertaking tertiary education and one had a full-time job, suggesting that their conditions were being adequately controlled. Half the caregivers were males, as were more than half the clients, indicating that men both seek help and support those who were ill.

### Thematic

The study shows that appropriate use of mental health service is hampered by physical, financial and cultural barriers.

### Physical barriers

The study shows that the public is still only vaguely aware of mental disorders, the availability of mental health service and effective treatment outcomes. This is similar to previous study [20] conducted in the Yoruba speaking region of Nigeria, which shows the knowledge of mental disorders was generally poor, with only a minority of the study sample believing it is possible to successfully treat mental disorders in the hospital. Research in other developing countries shows increasing public understanding of mental health conditions and awareness that effective treatment is available is important [1, 16]. Gureje and Alem [19] argue that public education should be given prominence by policies so as to support affected families. It is necessary for people to know about what services are available and what can be expected from them, as well as identify when to seek professional support.

The long waiting times at the hospital makes access to mental health service very difficult and resulted in some service users giving up without receiving the necessary care. This may partly explain why approximately 70% of mental health services in Nigeria are provided by religious and spiritual healers [25]. The absence of community-based mental health services burdens hospital-based health care putting additional pressure on already-stretched services, adversely affecting quality care [26]. Furthermore, Jerkins and colleagues [27] argues that If the public health burden of mental disorders is to be effectively addressed, it is necessary for policies to adopt a strategic approach that encompasses much more than just curative services for acutely ill people. The unavailability of mental health services in rural communities has left many families with no alternative but to use whatever is available. According to Mwape et al [28] Integrating mental health services into primary health care as a way of facilitating early detection and intervention for mental disorders is critical to improving and promoting the mental health of the population.

This study revealed that the long distances travelled to access mental health service created barriers for many service users, this being more common for rural dwellers that lack transportation, who had to take time away from their trade, work or home responsibilities. This finding is similar to another study of care-giving in this region [12], which showed that the distance travelled to access service was of a great concern and that as a result, many families could not sustain or continue with treatment. Furthermore, a study of burdens experienced by caregivers of patient with epilepsy in Northern Nigeria [11] indicated that this was mainly associated with their location of residence being outside the service catchment areas.

### Financial barriers

Service users experience financial burden in accessing mental health care, keeping follow-up appointments and paying for treatment. The need to pay for services results in many individuals being unable to sustain treatment, with the continuous use of prescribed medications being necessary to maintain an improved mental state. This finding is similar to a study on the burden of schizophrenia on rural and urban families in the same region [10], and showed that rural families experienced a heavy burden, mainly financial, as most are poor and have less disposable in-come to pay for transportation and treatment. A study of caregiver's burden and psychotic patient's perception of social support in Nigeria [29] showed that users without any form of social welfare net had higher burdens, experienced more family disharmony and poor illness outcomes. Evidence has shown that poverty and the absence of a social welfare net for mental health service users increases the burden of mental disorders, with poor mental health outcomes [1, 30, 31].

### Cultural barrier

Fear of stigma/discrimination was an important reason for not seeking or sustaining treatment due to the fear of what others may think, thereby preventing many from sustaining their treatments, finding being consistent with previous studies in Nigeria [20, 32]. The experiences associated with stigma/discrimination negatively impacted on their emotional/social well-being, resulting in families hiding ill relatives and not talk about it, which affected the families’ perceptions and ability about seeking appropriate help [15, 33]. It is important for society to understand how stigma impact on people with mental disorders and the need for change in public attitude.

### Limitation of the study

The authors are aware of the limitations associated with the sampling and sample bias as a result of a purposive sampling, and that the study was conducted in a single facility providing mental health service. Transferability is therefore limited, but as a first study in the Niger Delta region of Nigeria, the findings have implications that could influence both subsequent research, practice and policy innovations for care.

### Policy implications and recommendations

A number of recommendations are made to improve access to mental health services and improve service delivery. Information about what services are available and where, needs to be widely disseminated to encourage people to seek treatment when they find themselves experiencing the signs and symptoms of mental disorders. Services need to be expanded and the number of mental health professionals, increased to assist in the identification, management and prevention of symptoms at all levels of care. The challenges associated with centralised care need to be addressed, specifically the waiting times, access and the cost of services. Social welfare nets in the form of free medication or a subsidy for the low-income as a support mechanism would reduce the financial burden of many families and persons with mental illness, and allow them to sustain treatment. Social education needs to occur for people to overcome cultural barriers that impact on those with mental disorders and highlights the need for change in public attitude to support help seeking.

## Conclusion

This study shows that the centralised mental health service for a population of over four million is unable to provide the required services and support. Effective utilization of service needs to be supported by adequate resourced and staffed facilities that encourage good health seeking behaviour and sustain treatment follow-up. To overcome these barriers, priority should be given to mental health service delivery. Services should be provided throughout the health care system to enable people access them locally and affordably, preventing the need to travel and promoting service uptake and treatment continuation, only then will persons with mental disorders live productive and fulfilled lives.
